# Increasing Cattle Herd Size or Density in C_3_ Grass–Legume Meadows Reduces Dietary Quality

**DOI:** 10.3390/ani15020230

**Published:** 2025-01-16

**Authors:** John Derek Scasta, Fernando Forster Furquim

**Affiliations:** 1 Department of Ecosystem Science, Laramie Research and Extension Center, University of Wyoming, Laramie, WY 82072, USA; 2 Graduate Program in Botany, Universidade Federal do Rio Grande do Sul, Porto Alegre 91540-000, Brazil; ff.furquim@gmail.com

**Keywords:** *Bos taurus*, *Bromus inermis*, grazing management, livestock welfare, *Medicago sativa*, near-infrared reflectance spectroscopy, NIRS

## Abstract

Animal density is a grazing management decision implemented by managers that may reduce animal diet quality. Over three years, we collected herd-level cattle samples with varying animal numbers and densities across C_3_ grass–legume meadows near Powell, WY, USA. Paddocks ranged in size from 3 to 72 ha, cattle groups ranged from 80 to 370 animals, animal units (AUs; defined as heifers = 0.8, cow–calf pairs = 1.3, and bulls = 1.6) ranged from 52 to 248.6, animal density ranged from 1.5 to 30.3 animals/ha, and AU density ranged from 0.9 to 19.8 AUs/ha. Near-infrared reflectance spectroscopy of fecal samples (f.NIRS) was used to estimate crude protein (CP), digestible organic matter (DOM), and the DOM:CP ratio. Structural equation modeling (SEM) that mediated time (i.e., Day of Year) rendered all animal number or density variables as significant predictors for CP and DOM (negative estimates) and for DOM:CP (positive estimate), indicating that congregating animals in larger groups and/or greater densities, even in meadows with high-quality forage species, may reduce post-ingestive diet quality estimates by −1.2% and −2.0 for CP and DOM for every +100 animals or for every +10 animals per hectare. Livestock managers could potentially override this negative effect by adjusting when animals are congregated at higher densities.

## Simple Summary

Animal density is a grazing management decision implemented by managers that may reduce animal diet quality. Over three years, we collected herd-level cattle samples with varying animal numbers and densities across cool-season (C_3_) grass–legume meadows near Powell, WY, USA. Paddocks ranged in size from 3 to 72 ha, cattle groups ranged in absolute number from 80 to 370 individual animals, group types were variable (including heifers, cows with calves, and heifers and cows with calves, and all groups included bulls for breeding), animal units ranged from 52 to 248.6, animal density ranged from 1.5 to 30.3 animals/ha, and AU density ranged from 0.9 to 19.8 AUs/ha. Modeling that mediated time (i.e., Day of Year) rendered all animal number or density variables as significant predictors with negative estimates for crude protein and digestible organic matter fecal-derived estimates indicating that congregating animals in larger groups and/or greater densities, even in meadows with high-quality forage species, may reduce cattle diet quality.

## 1. Introduction

Perennial grasslands are critical for ruminant agricultural production [1] and also provide multiple ecosystem services including habitat for grassland birds, soil stability, and carbon sequestration [2]. When these grasslands include legumes, such ecosystem services that are affected include soil organic carbon storage [3], soil nitrogen transfer to grasses [4], and pollen and nectar resources for pollinators [5]. Forage legumes also are important for ruminant production systems, given the symbiotic relationship with nitrogen-fixing bacteria, which can benefit the soils but also offer an enhanced quality of plant tissue for grazing animals [6].

For cattle, specifically, optimizing nutrition has many implications for individual animals, herds, and ranching enterprise viability. From an animal health perspective, as the nutritional quality declines, there is an associated reduction in immune function, particularly during stressful periods [7]. From an animal performance perspective, nutritional quality influences animal weight and the deposition of adipose tissue, which is reflected in body condition, with implications for reproductive females in terms of lifetime fertility and the time to re-conception postpartum [8]. Mechanistically, nutrition also affects reproductive factors directly, including metabolic hormones, ovarian follicles, oocytes, and embryos [8]. Balancing multiple indicators of forage quality is also important for optimizing rumen digestion and efficiency, particularly the synchrony of forage-derived protein and carbohydrates [9]. Pastures that include cool-season C_3_ grasses and legumes, which can be dynamic through space and time, may offer optimal nutritional resources due to the influence on ruminant nitrogen efficiency as affected by energy-to-protein ratios [10]. In addition to the composition or volume of the forage sward, the management of grazing including intensity, duration, and timing also influences inter-animal competition, selection, and animal nutrition and performance, with implications for environmental sustainability if the volume of forage is over-depleted [11].

Nevertheless, linking animal diet quality with forage and environmental characteristics has been a persistent challenge, and recent non-invasive technologies offer insights previously unavailable [12]. In particular, the application of the near-infrared reflectance spectroscopy (NIRS) of feces (f.NIRS) offers an indirect and non-invasive method of estimating cattle diet quality relative to a variety of environmental or managerial variables [13,14]. Diet nutrition indicators estimated with f.NIRS include crude protein (CP), which is a function of mineral nitrogen and digestible organic matter (DOM), which is a reflection of energy [14]. In addition, the ratio of DOM:CP has been suggested to be a useful index of ruminal fermentation with an optimum range between 4.0 and 7.0, where higher values (>7.0) suggest potential protein deficiency and would be associated with mature dry forage conditions and lower values (<4.0) would be associated with lush green growing conditions [15].

The use of f.NIRS as a post-ingestive indicator of diet quality has shown that grazing management decisions, particularly the congregation of cattle into larger groups with more rapid rotations through pastures, may reduce the f.NIRS-predicted diet quality of yearling steers in the shortgrass steppe [16]. However, information is limited for how cow–calf pairs in different ecosystems may respond to such grazing management changes, which could have different implications for the cattle industry. Such information is particularly lacking for meadows with high-quality forage species. Moreover, the confounding influence of plant phenology, a well-established driver of forage quality for both improved forages [17] and native rangeland species [18], can make it difficult to untangle the effects of management. We therefore sought to quantify the f.NIRS-predicted dietary quality of cow-calf pairs in meadows dominated by cool-season C_3_ grasses such as smooth brome (*Bromus inermis*) and orchardgrass (*Dactylis glomerata*) and legumes such as alfalfa (*Medicago sativa*) in a North American intermountain basin relative to the management of herd size and density. We hypothesized that the biological mechanisms of animal selectivity and inter-animal competition would lead to reductions in dietary quality as the herd size or animal density increases.

## 2. Materials and Methods

### 2.1. Study Area

The research area is located in the 18b ecoregion known as the ‘the Bighorn basin’ which is an arid depression in the rain shadow of three mountain ranges including the Beartooth mountain range, the Absaroka mountain range, and the Pryor mountain range. The research property (44° 41′ 15.54″ N, 109° 00′ 53.70″ W) is owned by The Nature Conservancy (TNC) and is managed for multiple ecosystem services including the conservation of biodiversity.

### 2.2. Animal Management and Sampling

Cattle were *Bos taurus* primarily of Angus breed owned by two local ranchers. Cattle arrive each year in early June and stay until the fall. During the annual growing seasons (June through August) over three years (2017 to 2019), we collected 41 herd-level cattle samples with varying animal numbers and animal densities across various paddocks in four to six meadows annually near Powell, WY, USA. All grazing management decisions were made by the TNC cattle manager and, therefore, are considered a result of this study rather than an imposition of treatments. Animal research was approved by the University of Wyoming—Institutional Animal Care and Use Committee (IACUC) under protocol 20170713DS00275. We calculated paddock size, absolute animal numbers in each management group, and total number of animal units in each group (AUs; defined as heifers = 0.8, cow–calf pairs = 1.3, and bulls = 1.6), and then calculated animal or AU density relative to paddock size. Cattle diet quality was estimated post-ingestively using near-infrared reflectance spectroscopy (f.NIRS) of herd-level fecal samples for predictions of crude protein (CP), digestible organic matter (DOM), and the DOM:CP ratio with methods described by Craine et al. (2010) and Tolleson et al. (2020) [15,19] at the Texas A&M University—Grazing Animal Nutrition Laboratory. Across multiple paddocks annually (6 in 2017 and 4 in 2018 and 2019), before first cattle entrance, eighteen 1 m^2^ (1 m × 1 m) quadrats were systematically arranged along a grid, with distance of 20 m between them. In each quadrat, all plant species were identified to lowest possible taxonomic level and unknown specimens were collected and later identified using Dorn (2001) [20]. Cover of each plant species was estimated using modified Braun–Blanquet scale (i.e., <1%; 1–5%; 5–15%; 15–25%; 25–50%; 50–75%; and 75–100%). Vegetation surveys were conducted from June to August depending on cattle management during growing season. Environmental variability which could affect forage quality could include variability in soils, temperature, and moisture, although these were generally minimized with the selection of irrigated meadows for our study.

### 2.3. Statistical Analyses

We summarized the botanical data as the relative proportion of cool-season C_3_ grasses and legumes using descriptive statistics. For grazing management data which were determined independently by the manager, we calculated descriptive statistics (means and ranges [minimum and maximum]) of paddock size, cattle group size, animal units per group, animal density, and AU density. We also assessed histograms of each in order to understand distribution of the data.

**Figure 1:**
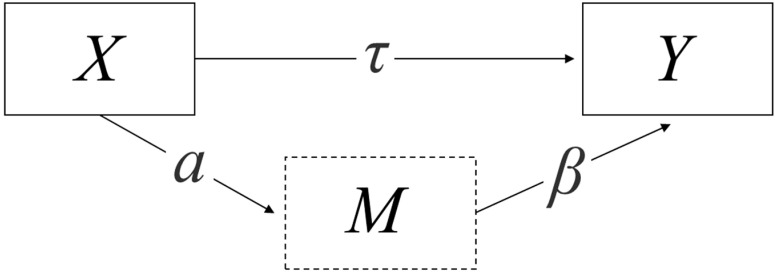
Theoretical structural equation model (SEM) using mediation analysis, where *X* are independent predictor management variables, *Y* are dependent nutrition response variables, *M* is the mediating variable considered as Day of Year, *τ* is the direct effect of *X* on *Y*, and *a* → *β* is the indirect effect where the total effect is the sum of the direct and indirect effect. Adapted from Biesanz et al. 2010 [22].

The f.NIRS dietary predictions of interest included CP and DOM, which are expressed as a percent and were, therefore, converted to proportions and then arcsine-transformed for analyses in order to meet assumptions of normality and heteroscedasticity. We then used linear mixed models to compare annual means of CP, DOM, and DOM:CP ratio with pasture as a random effect, and none were significantly different across years (all *p*-values > 0.05) using R code in the JASP LMM module [21]. Because there was no year effect, all future analyses assessed CP, DOM, and DOM:CP across years. To determine if there was a temporal effect of Day of Year on CP, DOM, and DOM:CP, and to further justify additional analyses, we applied linear least squares regressions across years for CP, DOM, and DOM:CP and assessed the correlation coefficient, *p*-values at the 95% confidence level, Shapiro–Wilks test for normality, and a constant variance test. We then used structural equation modeling (SEM) to test # of Animals, # AUs, Animal Density (Animals/ha), and animal unit density (AUs/ha) using an SEM mediation analysis to hold Day of Year as the mediator and the 4 animal number or density variables (described above) as the predictors for CP, DOM, and DOM:CP (Figure 1) based on lavaan R package for structural equation modeling through the JASP SEM module [22,23]. Finally, we used parameter estimates and 95% confidence intervals to provide graphical visualizations of the mediated effects of the 4 animal number or density variables (again described above) based on three-year mean f.NIRS assumptions and within the ranges of variabilities of all of our sampled variables.

## 3. Results

**Figure 2:**
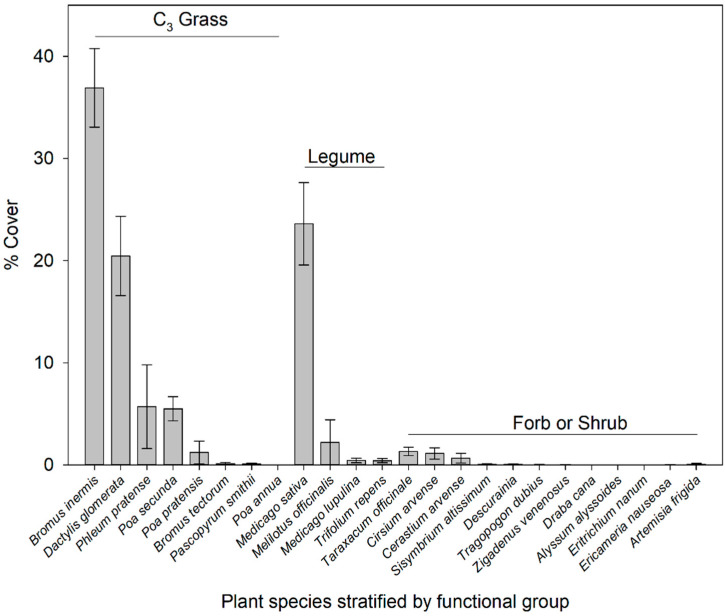
Abundance of plant species organized by dominance and functional groups (C_3_ Grass, Legume, and Forb/Shrub) in meadows at the TNC Heart Mountain Ranch Preserve near Powell, WY, USA.

Meadows were dominated by C_3_ grasses with a mean of 70% and a range of 48% to 96% across meadows and years, while legumes were the next most dominant with a mean of 27% and a range of 2% to 50%. Forbs were of low abundance with an average of 3% and a range of <1% to 9%. Species abundances organized by order from most to least within functional groups (C_3_ Grass > Legume > Forb/Shrub) are displayed in Figure 2. The dominant C_3_ grasses were *Bromus inermis* (mean of 37%), *Dactylis glomerata* (mean of 21%), *Phleum pratense* (mean of 6%), and *Poa secunda* (mean of 6%). All other grass species were less than 2% and included *Bromus tectorum*, *Pascopyrum smithii*, *Poa annua*, and *Poa pratensis*. The dominant legumes were *Medicago sativa* (mean of 24%), *Melilotus officinalis* (mean of 2%), *Medicago lupulina* (<1%), and *Trifolium repens* (<1%). Only three forbs had cover greater than 5% at any time and included *Cerastium arvense* (maximum of 5.4%), *Cirsium arvense* (maximum of 5.1%), and *Taraxacum officinale* (maximum of 5.2%). One shrub species and one sub-shrub species were detected in only one of the meadows and only at very low abundances, <1% (*Ericameria nauseosa* and *Artemisia frigida*, respectively).

**Figure 3:**
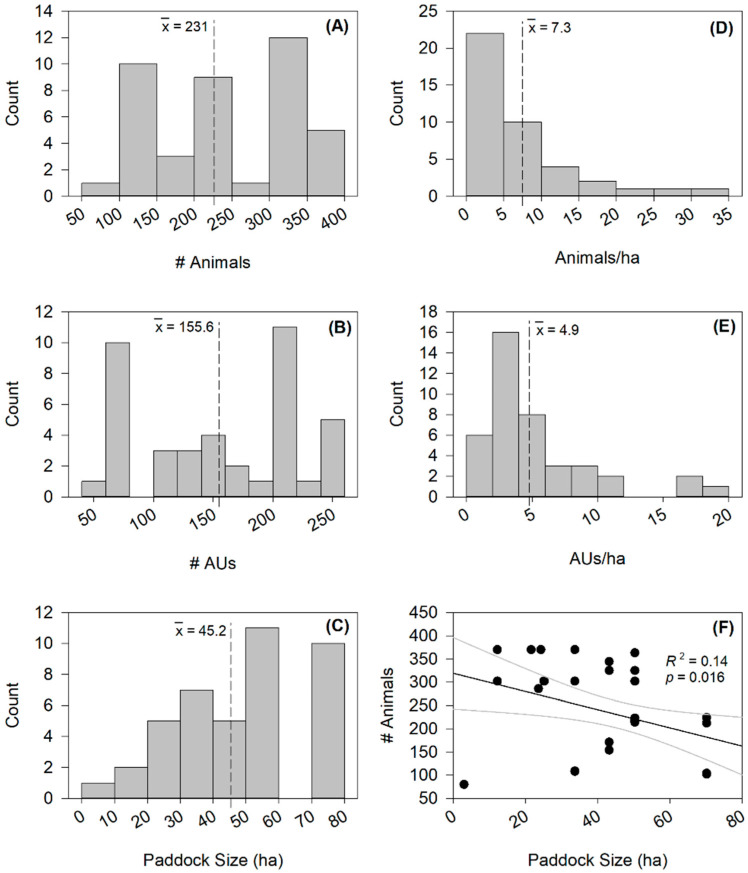
Histograms of grazing management data using mixed groups of breeding cattle (cows and calves, heifers, and bulls) including (**A**) number of animals, (**B**) number of animal units (AUs; defined as heifers = 0.8, cow–calf pairs = 1.3, and bulls = 1.6), (**C**) paddock size, (**D**) animal density defined as animals per hectare, (**E**) AU density defined as AUs per hectare, and (**F**) assessment of relationship between paddock size and number of animals in each management group.

The mean cattle groups had 231 individual animals and ranged from 80 to 370 individual animals (Figure 3A). The group types were variable (including heifers, cows with calves, and heifers and cows with calves, and all groups included bulls for breeding). The mean animal units per group were 155.6 and ranged from 52 to 248.6 (Figure 3B). The mean paddock size was 45.2 ha and ranged in size from 3 to 72 ha (Figure 3C). The mean animal density was 7.3 animals/ha and ranged from 1.5 to 30.3 animals/ha (Figure 3D). The mean AU density was 4.9 AUs/ha and ranged from 0.9 to 19.8 AUs/ha (Figure 3E). The numbers of animals and animal units in each respective group were evenly distributed (Figure 3A,B) across the gradient. The paddock size was skewed to the right with more large paddocks than small paddocks (Figure 3C). The animal density was skewed to the left with more low densities than high densities (Figure 3D). The AU density was slightly skewed to the left, but less so than animal density (Figure 3E). There was no relationship between the paddock size and number of animals in each group (Figure 3F).

**Figure 4:**
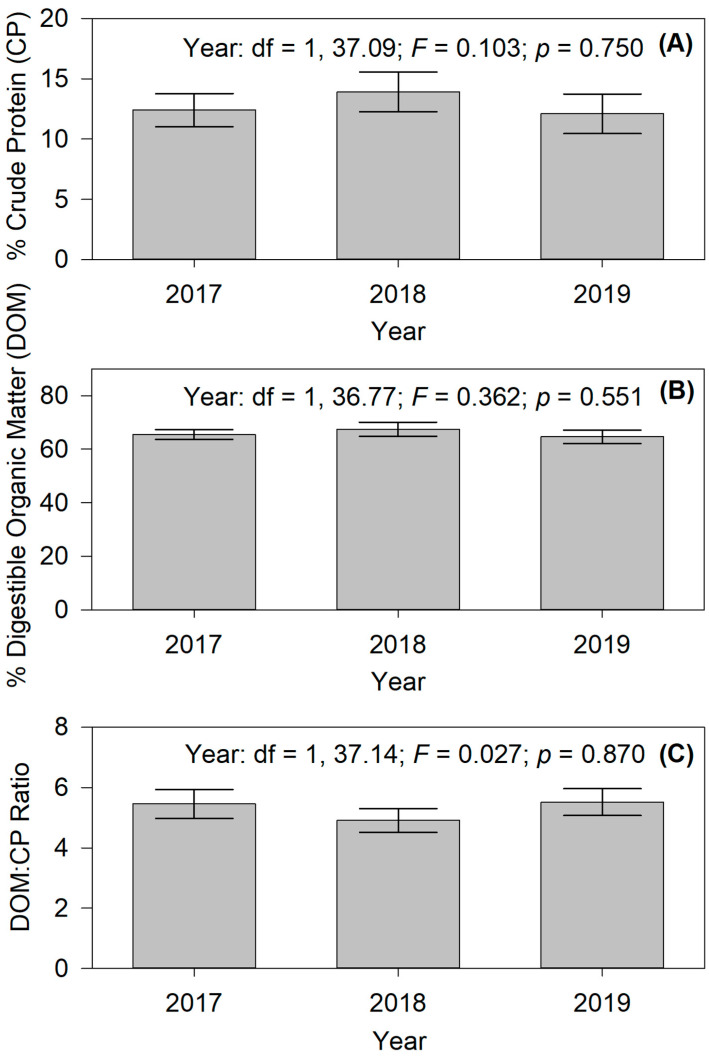
Bar graphs showing annual means and ±95% confidence intervals for (**A**) crude protein (CP), (**B**) digestible organic matter (DOM), and (**C**) the ratio of DOM:CP as indicators of animal nutrition and predicted by fecal near-infrared reflectance spectroscopy (f.NIRS).

Crude protein was on average 12.63% (±0.42% SE) across years, with no difference (df = 1, 37.09; F = 0.103; *p* = 0.750) between years (Figure 4A). Digestible organic matter was on average 65.62% (±0.63% SE), with also no difference (df = 1, 36.77; F = 0.362; *p* = 0.551) between years (Figure 4B). The DOM:CP ratio was on average 5.36 (± 0.13), with also no difference (df = 1, 37.14; F = 0.02; *p* = 0.870) between years (Figure 4C).

**Figure 5:**
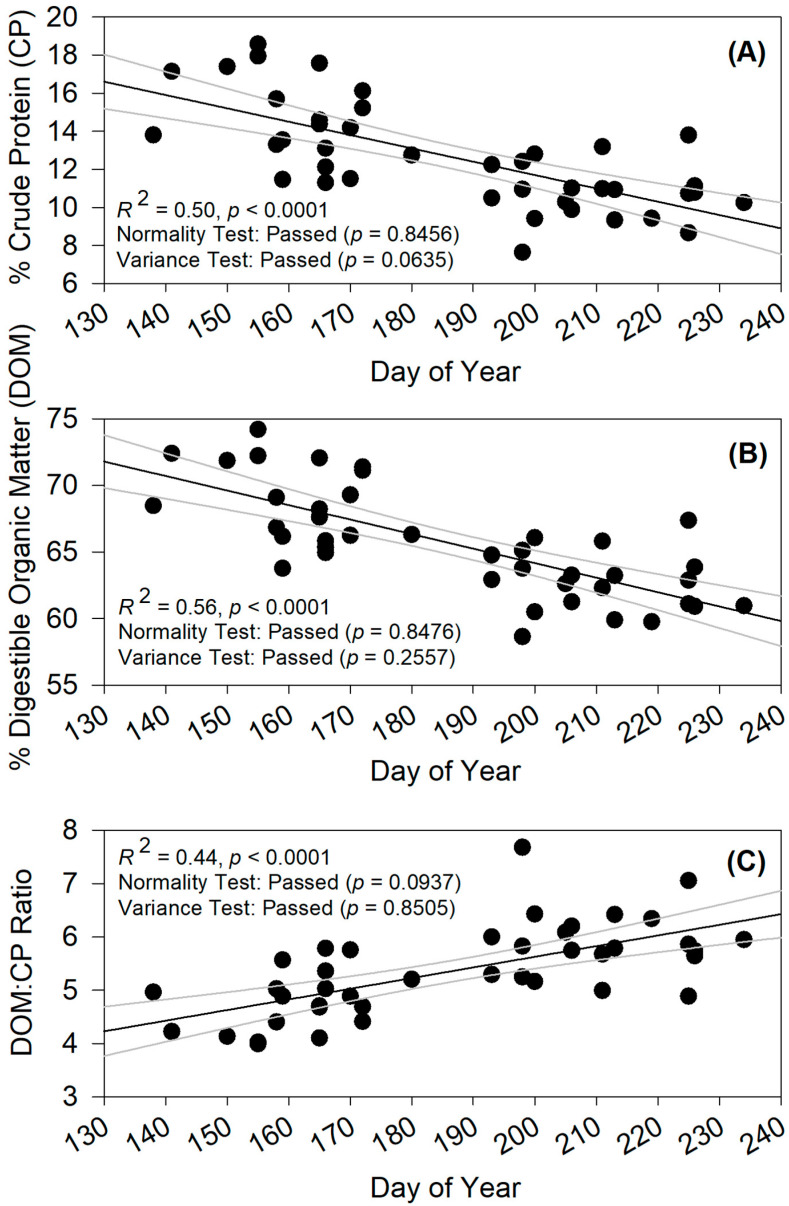
Temporal effect of time (Day of Year) for (**A**) crude protein (CP), (**B**) digestible organic matter (DOM), and (**C**) the ratio of DOM:CP demonstrated with linear least squares regression line graphs displaying fit linear trend lines and ±95% confidence intervals. CP, DOM, and DOM:CP were predicted using fecal near-infrared reflectance spectroscopy (f.NIRS).

The influence of time, as indicated by Day of Year, was significant for CP, DOM, and the DOM:CP ratio. CP declined through the growing season as it was significantly and negatively correlated with Day of Year (R^2^ = 0.50; *p* < 0.0001; Figure 5A). Similarly, DOM declined through the growing season as it was significantly and negatively correlated with Day of Year (R^2^ = 0.56; *p* < 0.0001; Figure 5B). The DOM:CP ratio had a different response to Day of Year as it increased through the growing season, as it was significantly and positively correlated with Day of Year (R^2^ = 0.44; *p* < 0.0001; Figure 5C). All three variables passed the Shapiro–Wilk test for normality and the constant variance tests (Figure 5A–C). The significant influence of Day of Year further justifies analytical techniques to mediate the temporal driver in order to better understand management influences.

**Figure 6:**
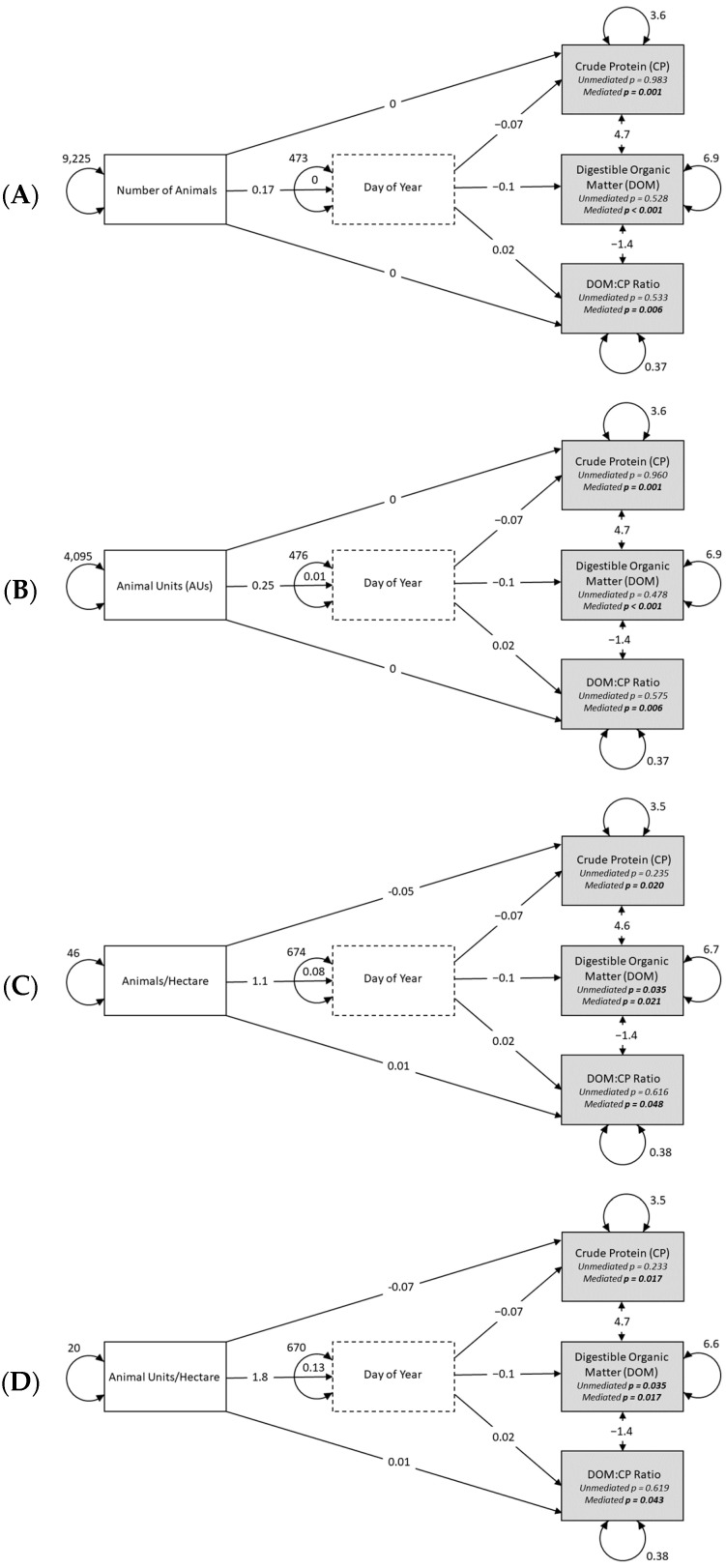
Path plots from structural equation modeling using mediation analyses for (**A**) number of animals, (**B**) number of animal units (AUs; defined as heifers = 0.8, cow–calf pairs = 1.3, and bulls = 1.6), (**C**) animal density defined as animals per hectare, (**D**) AU density defined as AUs per hectare, as mediated by day of year and for fecal near-infrared reflectance spectroscopy (f.NIRS)-predicted crude protein (CP), digestible organic matter (DOM), and DOM:CP.

**Figure 7:**
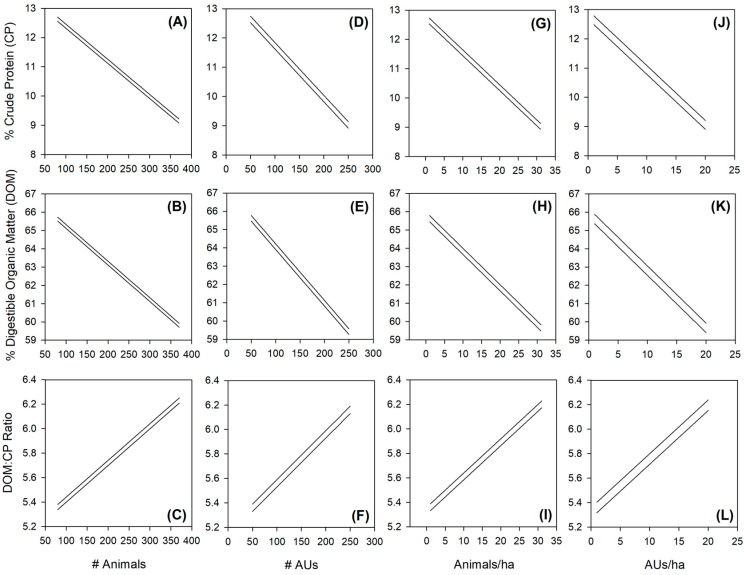
Graphical visualizations of the mediated effects of the 4 animal number or density variables [(**A**–**C**) number of animals, (**D**–**F**) number of animal units (AUs; defined as heifers = 0.8, cow–calf pairs = 1.3, and bulls = 1.6), (**G**–**I**) animal density defined as animals per hectare, and (**J**–**L**) AU density defined as AUs per hectare] based on three-year mean assumptions and within the ranges of variabilities of all of our sampled variables using SEM-calculated parameter estimates and 95% confidence intervals (where the two lines represent the upper and lower confidence interval that bracket the estimated mean). The top row of panels displays crude protein (CP), the middle row of panels displays digestible organic matter (DOM), and the bottom row of panels displays the DOM:CP ratio. CP, DOM, and DOM:CP were derived from fecal near-infrared reflectance spectroscopy (f.NIRS) predictions.

The SEM for the number of animals clarified the influence the size of the herd can have for f.NIRS predictions of diet quality when mediating for Day of Year. Specifically, the unmediated direct effects for CP, DOM, and DOM:CP were all non-significantly predicted by animal numbers (all *p*-values > 0.5 and all 95% CIs overlap zero) until Day of Year was dealt with as a mediating variable, at which point all *p*-values became significant (all *p*-values < 0.05 and no 95% CIs overlap zero) (Table 1; Figure 6A). Raw estimates confirm that there is a negative relationship for CP and DOM (−0.012 and −0.020, respectively) as the number of animals increases and a positive relationship for DOM:CP (0.003) as the number of animals increases (Table 1). For every increase of 100 animals, this would equate to a reduction in CP and DOM by 1.2% and 2.0%, respectively, and an increase in the DOM:CP ratio of 0.3 (Figure 7A–C).

The SEM for the number of animal units (AUs) clarified a similar influence as the number of animals for f.NIRS predictions of diet quality when mediating for Day of Year. Specifically, the unmediated direct effects for CP, DOM, and DOM:CP were all non-significantly predicted by AUs (all *p*-values > 0.4 and all 95% CIs overlap zero) until Day of Year was dealt with as a mediating variable, at which point all *p*-values became significant (all *p*-values < 0.05 and no 95% CIs overlap zero) (Table 1; Figure 6B). Raw estimates confirm that there is a negative relationship for CP and DOM (−0.018 and −0.031, respectively) as the number of AUs increases and a positive relationship for DOM:CP (0.004) as the number of AUs increases (Table 2). For every increase of 100 AUs, this would equate to a reduction in CP and DOM by 1.8% and 3.1%, respectively, and an increase in the DOM:CP ratio of 0.4 (Figure 7D–F).

The SEM for the density of animals (animals per hectare) clarified a somewhat different influence compared to the number of animals or number of AUs for f.NIRS predictions of diet quality when mediating for Day of Year. Specifically, the unmediated direct effects for CP and DOM:CP were both non-significantly predicted by the density of animals (all *p*-values > 0.2 and both 95% CIs overlap zero) until Day of Year was dealt with as a mediating variable, at which point those *p*-values became significant (all *p*-values < 0.05 and no 95% CIs overlap zero) (Table 3; Figure 6C). The exception here was for DOM which was significant in the unmediated analysis (*p* = 0.035 and 95% CIs did not overlap zero; Table 3). Raw estimates confirm that there is a negative relationship for CP and DOM (−0.120 and −0.199, respectively) as the density of animals increases and a positive relationship for DOM:CP (0.028) as the density of animals increases (Table 3). For every increase of 10 animals per hectare, this would equate to a reduction in CP and DOM by 1.2% and 2.0%, respectively, and an increase in the DOM:CP ratio of 0.3 (Figure 7G–I).

The SEM for the density of AUs (AUs per hectare) was similar to that for the density of animals for f.NIRS predictions of diet quality when mediating for Day of Year. Specifically, the unmediated direct effects for CP and DOM:CP were both non-significantly predicted by the density of animals (all *p*-values > 0.2 and both 95% CIs overlap zero) until Day of Year was dealt with as a mediating variable, at which point those *p*-values became significant (all *p*-values < 0.05 and no 95% CIs overlap zero) (Table 4; Figure 6D). The exception here again was for DOM which was significant in the unmediated analysis (*p* = 0.035 and 95% CIs did not overlap zero; Table 4). Raw estimates confirm that there is a negative relationship for CP and DOM (−0.188 and −0.313, respectively) as the density of animals increases and a positive relationship for DOM:CP (0.044) as the density of animals increases (Table 4). For every increase of 10 AUs per hectare, this would equate to a reduction in CP and DOM by 1.9% and 3.1%, respectively, and an increase in the DOM:CP ratio of 0.4 (Figure 7J–L).

## 4. Discussion

The results reported herein show that there is a generalizable effect of decreasing diet quality as the cattle herd size or density increases. This effect has been shown in other ecosystems, from arid and semi-arid rangelands to wetlands, for various types of grazing livestock including horses, beef cows, yearling cattle, and sheep [11,16,24]. Specifically for arid environments, similar results were found in the North American saltbush rangelands where sheep consumed less nutritious diets with heavier utilization [11]. Specifically for semi-arid environments, similar results were found in the North American shortgrass steppe where adaptive multi-paddock grazing using 10× larger groups of yearling cattle reduced the diet quality [16]. Specifically for wetlands, similar results were found in the Netherlands in wetlands where cattle and horses both had a lower diet quality and body condition when the herbivore density was higher [24]. Our results add another environmental insight to this established knowledge that suggests the effect of animal numbers or density in reducing diet quality even in high-quality C_3_ grass–legume meadows, which are different than arid and semi-arid rangelands or wetlands. This effect is likely an outcome of the increasing inter-animal competition, or, in other words, the more animals are congregated in a discrete area, the more they are in competition to satisfy their nutritional requirements. Bulk roughage intake feeders such as cattle [25] and other large bovids such as bison are likely balancing their time (attempting to minimize this) and energy intake (attempting to maximize this) [26].

Nevertheless, when animals perceive other animals in their cohort may be getting ahead of them during grazing, they may feel compelled to eat more quickly and less selectively, resulting in a lower-quality diet—a phenomena that manifests as herd size or density increases [24]. The mechanism driving this effect is a functionally less efficient bite process where animals may have less volume per bite as the animal density increases with alterations in plant part or species selection [27]. Such selection alterations can lead to reduced diet quality parameters such as crude protein [28] or in vitro digestibility [29]. Specifically, a reduction in grass composition and an increase in shrub composition of heifer cattle diets as the stocking rate increased in a forested rangeland have also been reported [29]. This effect has been observed and manipulated by ranchers [30] as well as shown in other places beyond North America such as Mediterranean grasslands where cows had a lower diet quality at higher densities during the dormant season [31].

Another important result from this study is that managers can use different indicators of animal density including the absolute number of animals in the herd, the number of animals per unit area, or the animal unit equivalent per unit area—all of which had significant and negative relationships with diet quality [30]. For example, they may note that, for every +100 animals or for every +10 animals per hectare, they can anticipate a −1.2% and −2.0 decrease for CP and DOM, respectively.

The use of the SEM offers an opportunity to mediate known drivers of diet quality, in our case, seasonality and plant phenology, to more clearly isolate managerial drivers. Managers may be able to use such information by deciding when it is optimal to congregate animals in larger groups or not. From a rotational grazing scenario perspective, managers may decide to congregate animals in larger groups (or smaller paddocks) earlier in the growing season when the forage quality is higher but then disperse animals into smaller groups (or larger paddocks) later in the growing season when the forage quality is lower. Managers may also consider altering paddock designs and sizes when considering animal numbers accordingly. Future research should further incorporate fecal samples with forage samples through the growing season and across a gradient of manager decisions as applied in our experiment to further elucidate such interactions and possibilities. An additional area of research could include the scenario above coupled with enteric methane (CH_4_) emissions measurements from animals in order to understand if adjustments to grazing management decisions could achieve reductions [32].

## 5. Conclusions

Livestock managers should incorporate our results that show how herd size. independently, and relative to paddock size and animal density, can affect diet quality even in meadows with palatable C_3_ grasses and legumes [33]. Practically, this could be used to enhance animal nutrition during critical production periods or manipulate utilization for certain vegetation management objectives [13]. Advanced diet quality estimation methods, such as f.NIRS [34], coupled with advanced analytical techniques such as SEMs, will continue to be important for refining animal nutrition in extensive pasture and rangeland environments and for facilitating complex transdisciplinary research [35]. These advances have implications for animal welfare and performance and could help inform the management of greenhouse gas emissions such as CH_4_, which will be important given the increase in societal scrutiny and consumer choice change.

## Figures and Tables

**Table 1:** Results from structural equation model using mediation analysis for unmediated direct effects and total effects of number of animals on fecal near-infrared reflectance spectroscopy (f.NIRS)-predicted crude protein (CP), digestible organic matter (DOM), and DOM:CP as mediated by Day of Year (DoY).

Unmediated Direct Effects	Estimate ^1^	Estimate ^2^	SE	*z*-Value	*p*-Value	95% CIs
# Animals → CP	1.195 × 10^−6^	−3.852 × 10^−4^	5.646 × 10^−5^	0.021	0.983	−1.095 × 10^−4^ to 1.119 × 10^−4^
# Animals → DOM	−3.626 × 10^−5^	−0.003	5.750 × 10^−5^	−0.631	0.528	−1.190 × 10^−4^ to 7.644 × 10^−5^
# Animals → DOM:CP	−7.888 × 10^−4^	−7.888 × 10^−4^	0.001	−0.623	0.533	−0.003 to 0.002
**Mediated Total Effects**						
# Animals → DoY → CP	−1.737 × 10^−4^	−0.012	5.358 × 10^−5^	−3.242	0.001	−2.787 × 10^−4^ to −6.868 × 10^−5^
# Animals → DoY → DOM	−2.165 × 10^−4^	−0.020	5.607 × 10^−5^	−3.861	<0.001	−3.264 × 10^−4^ to −1.066 × 10^−4^
# Animals → DoY → DOM:CP	0.003	0.003	0.001	2.739	0.006	8.062 × 10^−4^ to 0.005

^1^ Transformed estimate; ^2^ Raw estimate.

**Table 2:** Results from structural equation model using mediation analysis for unmediated direct effects and total effects of number of animal units (AUs; defined as heifers = 0.8, cow–calf pairs = 1.3, and bulls = 1.6) on fecal near-infrared reflectance spectroscopy (f.NIRS)-predicted crude protein (CP), digestible organic matter (DOM), and DOM:CP as mediated by Day of Year (DoY).

Unmediated Direct Effects	Estimate ^1^	Estimate ^2^	SE	*z*-Value	*p*-Value	95% CIs
# AUs → CP	−4.304 × 10^−6^	−0.071	8.624 × 10^−5^	−0.050	0.960	−1.733 × 10^−4^ to 1.647 × 10^−4^
# AUs → DOM	−6.233 × 10^−5^	−0.132	8.791 × 10^−5^	−0.709	0.478	−2.346 × 10^−4^ to 1.100 × 10^−4^
# AUs → DOM:CP	−0.001	0.010	0.002	−0.561	0.575	−0.005 to 0.003
**Mediated Total Effects**						
# AUs → DoY → CP	−2.632 × 10^−4^	−0.018	8.139 × 10^−5^	−3.234	0.001	−4.227 × 10^−4^ to −1.037 × 10^−4^
# AUs → DoY → DOM	−3.287 × 10^−4^	−0.031	8.489 × 10^−5^	−3.872	<0.001	−4.950 × 10^−4^ to −1.623 × 10^−4^
# AUs → DoY → DOM:CP	0.004	0.004	0.002	2.740	0.006	0.001 to 0.007

^1^ Transformed estimate; ^2^ Raw estimate.

**Table 3:** Results from structural equation model using mediation analysis for unmediated direct effects and total effects of number of animals per hectare (Animals/ha) on fecal near-infrared reflectance spectroscopy (f.NIRS)-predicted crude protein (CP), digestible organic matter (DOM), and DOM:CP as mediated by Day of Year (DoY).

Unmediated Direct Effects	Estimate ^1^	Estimate ^2^	SE	*z*-Value	*p*-Value	95% CIs
Animals/ha → CP	−6.126 × 10^−4^	−0.045	5.159 × 10^−4^	−1.187	0.235	−0.002 to 3.985 × 10^−4^
Animals/ha → DOM	−8.894 × 10^−4^	−0.084	4.227 × 10^−4^	−2.104	0.035	−0.002 to −6.085 × 10^−5^
Animals/ha → DOM:CP	0.006	−0.006	0.013	0.502	0.616	−0.018 to 0.031
**Mediated Total Effects**						
Animals/ha → DoY → CP	−0.002	−0.120	7.459 × 10^−4^	−2.320	0.020	−0.003 to −2.686 × 10^−4^
Animals/ha → DoY → DOM	−0.002	−0.199	9.136 × 10^−4^	−2.309	0.021	−0.004 to −3.192 × 10^−4^
Animals/ha → DoY → DOM:CP	0.028	0.028	0.014	1.974	0.048	2.006 × 10^−4^ to 0.056

^1^ Transformed estimate; ^2^ Raw estimate.

**Table 4:** Results from structural equation model using mediation analysis for unmediated direct effects and total effects of number of animal units per hectare (AUs/ha) on fecal near-infrared reflectance spectroscopy (f.NIRS)-predicted crude protein (CP), digestible organic matter (DOM), and DOM:CP as mediated by Day of Year (DoY).

Unmediated Direct Effects	Estimate ^1^	Estimate ^2^	SE	*z*-Value	*p*-Value	95% CIs
AUs/ha → CP	−9.597 × 10^−4^	−0.071	8.052 × 10^−4^	−1.192	0.233	−0.003 to 6.185 × 10^−4^
AUs/ha → DOM	−0.001	−0.132	6.639 × 10^−4^	−2.113	0.035	−0.003 to −1.018 × 10^−4^
AUs/ha → DOM:CP	0.010	0.010	0.020	0.497	0.619	−0.029 to 0.048
**Mediated Total Effects**						
AUs/ha → DoY → CP	−0.003	−0.188	0.001	−2.380	0.017	−0.005 to −4.795 × 10^−4^
AUs/ha → DoY → DOM	−0.003	−0.313	0.001	−2.386	0.017	−0.006 to −5.924 × 10^−4^
AUs/ha → DoY → DOM:CP	0.044	0.044	0.022	2.022	0.043	0.001 to 0.087

^1^ Transformed estimate; ^2^ Raw estimate.

## Data Availability

Data may be made available upon reasonable request.
